# Management of Failed Fixation of Extracapsular Hip Fractures

**DOI:** 10.7759/cureus.73208

**Published:** 2024-11-07

**Authors:** Nicholas A Platt, James Pegrum, Hamish Macdonald

**Affiliations:** 1 Trauma and Orthopaedics, Gloucestershire Royal Hospital, Gloucester, GBR; 2 Trauma and Orthopaedics, Gloucester Royal Hospital, Gloucester, GBR

**Keywords:** acute total hip replacement, cephalo-medullary nailing, dynamic hip screw fixation, extracapsular hip fracture, failed fixation, hip hemi-arthroplasty, neck of femur fracture, open reduction internal fixation, revision fixation

## Abstract

Background

Extracapsular hip fractures are routinely treated with fixation, and the majority heal without complication. The fixation fails in a minority of cases, typically either by ‘cutting out’ of the superior femoral head or through breakage of the metalwork following non-union. In such cases, if operative treatment is thought appropriate, there are two major treatment options: revision fixation of the fracture or joint replacement surgery.

Methods

Medline on OvidSP was searched using relevant medical-specific subject headings (MeSH) and keywords. The inclusion criteria were: study regarding management of failed extracapsular hip fracture fixation (not osteoarthritis alone, following such surgery), mean age >60 years, comparative study of joint replacement vs. revision fixation.

The search returned 1053 results, of which two were relevant. Both studies were considered poor quality and neither study was randomised. Instead, outcomes from the current Hospital Trust were used instead through a prospectively generated trauma database.

Results

From the trauma database, 37 patients (mean age 81), of whom 21 had received cephalomedullary nails and the remainder dynamic hip screw (DHS), were identified. Fourteen patients underwent revision fixation (seven cephalomedullary nail; seven blade plate), while 23 underwent hip replacement (17 total hip replacement; six hemiarthroplasty). Although the difference did not reach statistical significance according to the log-rank test (p = 0.233), there is a trend towards lower re-operation rate following joint replacement, with the difference becoming apparent after over one year’s follow-up.

Conclusion

Despite the quality of evidence, the default operation for failed extracapsular hip fracture fixation should be joint replacement, based on a likely lower re-operation rate and permitting immediate full-weight-bearing. In selected instances, particularly younger patients and those who can partially-weight-bear, revision fixation would still be considered. As the number of hip fractures continues to increase both within the UK and worldwide, we can expect to see more patients with previous failed fixations, and more evidence regarding the advantages and disadvantages of different treatment strategies is required.

## Introduction

Over 65,000 hip fractures occur in patients aged over 65 per year in the United Kingdom, and between a third and half are extracapsular [1,2]. These are typically managed with fixation, but in up to 5% of cases fixation fails [3]. Failure is either through ‘cut-out’ whereby the femoral head screw(s) penetrate the superior femoral head following fracture collapse, or by non-union of the fracture followed by metal fatigue and implant breakage. Treatment may include revision fixation or hip replacement [4,5]. Revision fixation preserves the native hip joint and is a smaller physiological insult but carries the risk of further cut-out or non-union. A period of restricted weight-bearing may also be required [6]. Replacement is potentially larger surgery, and carries specific risks particularly of dislocation, but permits early full weight-bearing and does not rely on bone healing [7,8].

## Materials and methods

Methods

Although mobility and quality of life are the most important factors to consider, we felt that studying re-operation rates would allow the question to be answered objectively. Further operations have a significant effect on patients so this approach was chosen [9-11].

**Figure 1:**
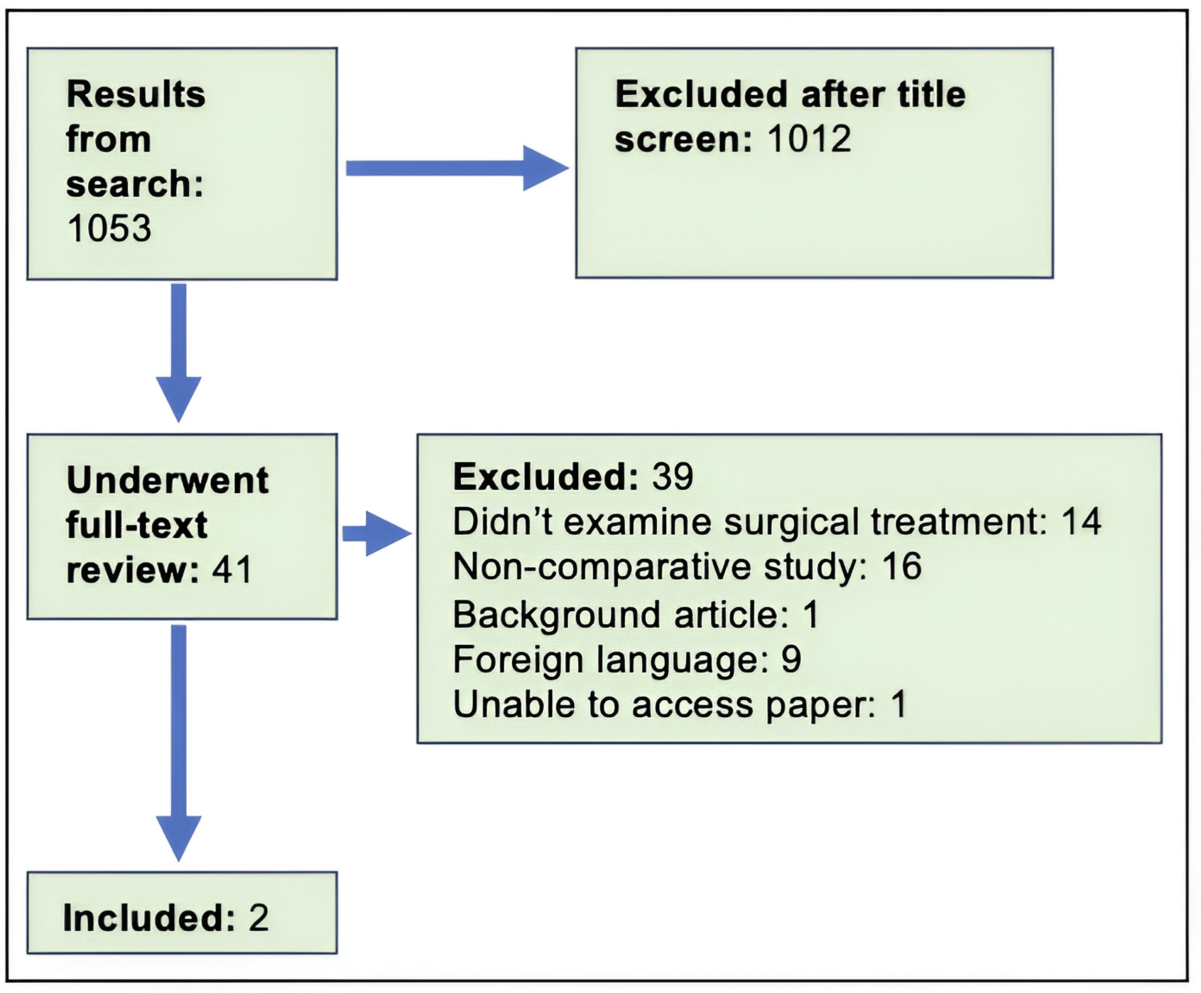
Literature search

Initially, a literature search was undertaken, demonstrated in Figure 1. Medline on OvidSP was searched using relevant medicine-specific headings (MeSH) and keywords. The inclusion criteria were: study regarding the management of failed extracapsular hip fracture fixation (not osteoarthritis alone, following such surgery), mean age >60 years, comparative study of joint replacement vs. revision fixation. As it was data from the already-present and routinely collected database, and it was purely the analysis of outcomes, without including confidential data, additional ethics committee approval was not required.

Only two studies were relevant. Both studies were considered poor quality and neither study was randomised. Therefore, it was decided to review patient outcomes from a local trust instead. Through retrospective analysis of a prospectively collated trauma database (January 1, 2014 to February 1, 2021), patients with failed extracapsular fracture fixations who underwent surgical management were identified. Baseline demographic data was collected, in addition to the type of surgical treatment performed (revision fixation or joint replacement), dates of surgeries, most recent follow-up, most recent imaging, further surgeries, mobility status and date of death where applicable.

The Stata software (StataCorp LLC, College Station, TX) was used for performing the Kaplan-Meier survival analysis, with re-operation for any cause as the endpoint. The study exit time was defined as either re-operation date or, if no re-operation had occurred, the later of the most recent clinical follow-up or most recent radiograph. Patients were also censored at death.

## Results

Local Trust trauma database

Thirty-seven patients (mean age 81 years, age range 58-93 years, standard deviation 8.3), of whom 21 had received cephalomedullary nails and the remainder dynamic hip screw (DHS) were identified. Fourteen patients underwent revision fixation (seven cephalomedullary nail; seven blade plate), while 23 underwent hip replacement (17 total hip replacement; six hemiarthroplasty). Table 1 outlines the treatment characteristics of the patients from the trauma database. Table 2 demonstrates the outcomes in patients that required further surgery.

**Table 1:** Baseline and treatment characteristics of identified patients. DHS: Dynamic hip screw; THR: total hip replacement

	n	Age (years)	Female (%)	Initial implant	Treatment detail
Revision fixation	14	80	79%	2 DHS, 11 Long nail, 1 blade plate	7 long nail, 7 blade plate
Joint replacement	23	81	74%	12 DHS, 9 long nail, 1 short nail, 1 locking plate	17 THR, 6 hemiarthroplasty
Total	37	81	76%	N/A	N/A

**Table 2:** Outcomes of identified patients. THR: Total hip replacement; ORIF: open reduction internal fixation; * = mean (range).

	Follow-up (months)*	Further surgery		Deaths
		n	Details	
Revision fixation	8.6 (0-42)	5	3 THR, 1 proximal femoral replacement, 1 washout	6
Joint replacement	14.3 (0-49)	4	2 ORIF periprosthetic fracture, 1 revision for dislocation, 1 excision arthroplasty	9
Total	12.1 (0-49)	9	N/A	15

Although there was a trend towards fewer re-operations with joint replacement, this was not statistically significant. The 95% confidence intervals widely overlapped, and the log-rank test gave a p-value of 0.23.

As the papers from literature review did not provide sufficient detail to perform meta-analysis or survival analysis, a chi-squared test was performed for re-operations between revision fixation and joint replacement, pooled across the published studies and the local trust database. There were 39 revision fixations, of which 10 (26%) underwent further surgery. Of the 52 joint replacements, eight (15%) underwent further surgery. Chi-squared test (Table 3) gave a p-value of 0.08 below the conventional threshold of statistical significance.

**Table 3:** Chi-squared for pooled data Chi-square statistic: 3.0586 p-value: 0.08031

	Re-operation	No further surgery	Marginal row totals
Revision fixation	10 (6.86) [1.44]	29 (32.14) [0.31]	39
Arthroplasty	6 (9.14) [1.08]	46 (42.86) [0.23]	52
Marginal column totals	16	75	91 (grand total)

**Figure 2:**
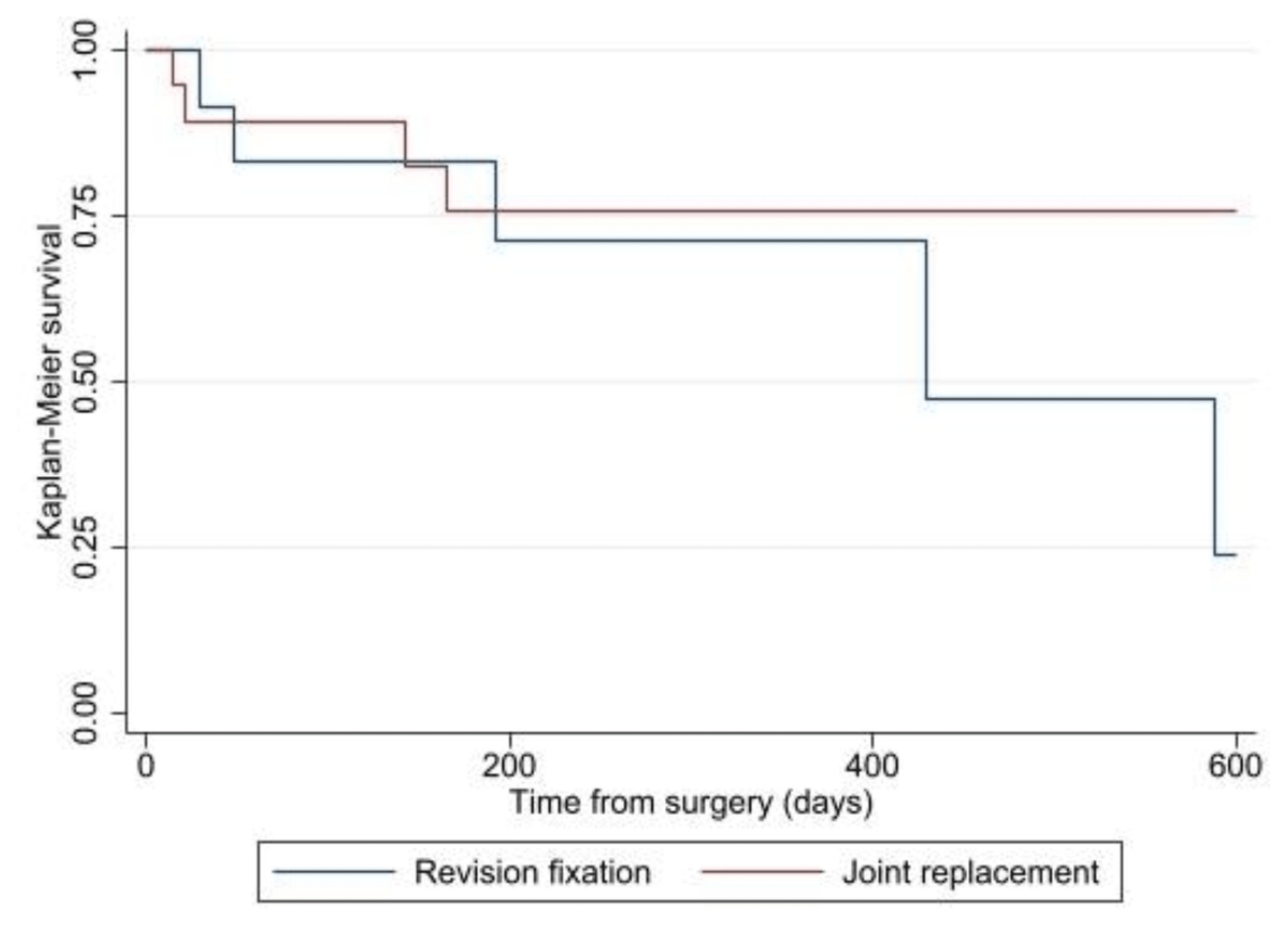
Kaplan-Meier survival analysis

The survival curve is demonstrated in Figure 2. Although the difference did not reach statistical significance according to the log-rank test (p = 0.233), there is a trend towards lower re-operation rate following joint replacement, with the difference becoming apparent after over one year’s follow-up.

Literature search

Brunner et al. [12] examined the salvage of failed cephalomedullary nail fixation (n=24, mean age 83 years) with revision nailing or joint replacement. The follow-up period was unclear, but two of three revision nail patients required re-operation, as opposed to one of the 21 joint replacement patients.

Said et al. [13] examined salvage of failed DHS fixation (n=26, mean age 61 years) with revision plate (DHS or blade plate) fixation ±subtrochanteric valgus osteotomy. After a mean follow-up period of 31 months, one of 18 revision fixation patients had required re-operation, as opposed to one of eight joint replacement patients.

## Discussion

Published literature is of low quality and the two comparative cohort studies give contradictory results. An analysis of local cases supports the use of joint replacement, but without reaching statistical significance. Of note, the cohort is heterogeneous and includes both cut-out and nonunion patients.

Decision-making in failed hip fracture fixation is challenging, with multiple occasionally competing elements. For example, the deleterious effects of prolonged non-weight-bearing are well known [14,15], and joint replacement might be more likely to allow immediate full-weight-bearing at the cost of a larger operation with increased blood loss and more monetary expense when compared to revision fixation. Ideally, one would take mortality, patient-reported outcomes (PROMs) and economic considerations into account when deciding on an optimal treatment, but literature search did not find any studies that examined differences in patient-reported outcome measures (PROMs) or economic outcomes between the two treatments.

We know that in primary hip fracture surgery re-operations are associated with increased mortality [16,17]. Examining the relative rates of subsequent re-operations therefore does help inform treatment choice. Although literature search did not provide information regarding post-operative weight-bearing status, anecdotal discussion with local surgeons suggests that immediate full weight-bearing is encouraged following all joint replacement operations, but that a majority of surgeons would limit weight-bearing following revision fixation surgery. Permitting immediate full-weight-bearing is a cornerstone of hip fracture surgery, and so this would again bias towards joint replacement as the default treatment of failed extracapsular hip fracture fixation.

Revision fixation should still be considered, especially in younger patients who are more likely to require future joint replacement revision, those who can partially-weight-bear and those with good bone quality and/or subtrochanteric non-union as opposed to femoral head cut-out.

Combining the results from this study with the two studies previously published, the overall re-operation rate for revision fixation is 23% and that of joint replacement 12%. A study with 80% power to detect that magnitude of difference at a confidence level of 95% would require 220 patients per group (assuming 20% loss to follow-up). Even if 5% of extracapsular hip fracture fixations fail, then each year only 1300 would be eligible for such a trial in the UK. Recruiting sufficient patients for a randomised trial given the inherent difficulties of recruiting patients to trauma studies would be a challenge (World Hip Trauma Evaluation (WHiTE) Four randomised only half of the eligible patients in their study centres) [18].

In addition, to minimise heterogeneity, we would suggest only including patients without cut-out of the lag screw through the femoral head, further reducing the number of patients available for inclusion. This was supported by a survey of subspecialty hip and trauma surgeons within the local trust; while the majority felt there was a degree of uncertainty regarding patients in whom the femoral head and acetabulum were largely intact. There was no equipoise regarding the cut-out group, for whom all consultants felt joint replacement was the best solution. A multicentre prospective cohort study might be able to recruit sufficient patients, but we would anticipate requiring over a year to do so, followed by two years of follow-up.

Regarding PROMs, we do not think that sufficient information regarding likely outcomes exists to perform a sample size calculation based on this outcome. If the above study was performed, secondary outcome measures should be mortality and PROMs. If this study is performed correctly, one would also prospectively discuss with a health economic analysis specialist to ensure that sufficient information was collected to allow an estimation of the relative cost and utility of the two treatment strategies, including perioperative/in-hospital costs and those borne by society as a whole (e.g. increased social care or inability of the patient or family to return to work if applicable).

## Conclusions

Despite the quality of evidence, and considering the points above, the evidence suggests that the default operation for failed extracapsular hip fracture fixation should be joint replacement, based on a likely lower re-operation rate and permitting immediate full-weight-bearing. In selected instances, particularly younger patients and those who can partially bear weight, revision fixation would still be considered. As the number of hip fractures continues to increase both within the UK and worldwide, we can expect to see more patients with previous failed fixations, and more evidence regarding the advantages and disadvantages of different treatment strategies is required.
